# Sensitivity of the S1 neuronal calcium network to insulin and Bay‐K 8644 in vivo: Relationship to gait, motivation, and aging processes

**DOI:** 10.1111/acel.13661

**Published:** 2022-06-19

**Authors:** Ruei‐Lung Lin, Hilaree N. Frazier, Katie L. Anderson, Sami L. Case, Adam O. Ghoweri, Olivier Thibault

**Affiliations:** ^1^ Department of Pharmacology and Nutritional Sciences University of Kentucky Lexington Kentucky USA; ^2^ Present address: Kaia Health New York New York USA

**Keywords:** ambulation, falls, GCaMP6, imaging, therapy, two‐photon

## Abstract

Neuronal hippocampal Ca^2+^ dysregulation is a critical component of cognitive decline in brain aging and Alzheimer's disease and is suggested to impact communication and excitability through the activation of a larger after hyperpolarization. However, few studies have tested for the presence of Ca^2+^ dysregulation in vivo, how it manifests, and whether it impacts network function across hundreds of neurons. Here, we tested for neuronal Ca^2+^ network dysregulation in vivo in the primary somatosensory cortex (S1) of anesthetized young and aged male Fisher 344 rats using single‐cell resolution techniques. Because S1 is involved in sensory discrimination and proprioception, we tested for alterations in ambulatory performance in the aged animal and investigated two potential pathways underlying these central aging‐ and Ca^2+^‐dependent changes. Compared to young, aged animals displayed increased overall activity and connectivity of the network as well as decreased ambulatory speed. In aged animals, intranasal insulin (INI) increased network synchronicity and ambulatory speed. Importantly, in young animals, delivery of the L‐type voltage‐gated Ca^2+^ channel modifier Bay‐K 8644 altered network properties, replicating some of the changes seen in the older animal. These results suggest that hippocampal Ca^2+^ dysregulation may be generalizable to other areas, such as S1, and might engage modalities that are associated with locomotor stability and motivation to ambulate. Further, given the safety profile of INI in the clinic and the evidence presented here showing that this central dysregulation is sensitive to insulin, we suggest that these processes can be targeted to potentially increase motivation and coordination while also reducing fall frequency with age.

## Graphical abstract

To characterize potential central mechanisms underlying age‐dependent changes in ambulatory behavior, we tested for the presence of S1 neuronal Ca^2+^ network dysregulation in vivo in young and aged F344 rats using state‐of‐the‐art imaging techniques. We report increased overall activity and connectivity of the network along with decreased locomotor stability in aged animals, suggesting Ca^2+^ dysregulation may be generalizable to other brain areas and engage modalities associated with ambulation.

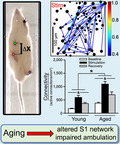

## 1 INTRODUCTION

Working exclusively in the field of hippocampal brain aging, our lab and others have identified L‐type voltage‐gated Ca^2+^ channels (L‐VGCCs) and Ca^2+^‐fluxing ryanodine receptors as contributors to the Ca^2+^ hypothesis of brain aging and Alzheimer's disease (AD) (Gant et al., 2006; Kumar & Foster, 2005; Moyer Jr et al., 1992; Norris et al., 1998; Oh et al., 2016; Stutzmann et al., 2006; Thibault et al., 2001). This work has provided insights into neuronal Ca^2+^‐dependent mechanisms responsible for age‐related cognitive and memory decline using mostly single‐cell imaging and electrophysiology techniques. However, despite years of progress, significant questions remain, including whether Ca^2+^ dysregulation is present in other brain areas, whether it occurs in vivo, and how this dysregulation contributes to neuronal communication within the network. To address this, we used in vivo imaging protocols to measure neuronal Ca^2+^ dynamics across hundreds of neurons in the cortex of young and aged rats, and investigated, for the first time, the Ca^2+^ hypothesis of brain aging in an area of the brain that has received little attention and is associated with ambulatory function.

The primary somatosensory cortex (S1) maps sensory discrimination and proprioception and is an integral component of movement. Superficial layers (layers 2/3) of S1, including local circuits, receive inputs from the thalamus and cortical areas associated with limb movement and sensory encoding (Cichon & Gan, 2015; Lacefield et al., 2019). Additionally, these layers also output to motor areas and the thalamus. Given that aging is positively correlated with fall frequency, that cautious gait and frailty are associated with vascular changes across large brain areas (Fasano et al., 2012), and that recent modeling data estimated a single intervention strategy could reduce the cost‐burden of patient care between $90 and $400 million (Stevens & Lee, 2018), the importance of investigating new, clinically relevant therapies targeting central modalities that are involved with sensorimotor encoding and locomotor function is clear. Despite this, basic research investigating age‐related degradation in these regions, such as neuronal circuits in the dorsomedial striatum or S1, is limited. For example, some studies have shown age‐dependent changes in excitability likely mediated by increased inhibitory post‐synaptic currents in deeper layers (layers 3–5) of S1, with no change in the slow afterhyperpolarization (AHP) (Hickmott & Dinse, 2013; Popescu et al., 2021). Recently, one study comparing AHP amplitudes between layers 2/3 and layer 5 S1 neurons in the C57BL/6 mouse did not provide evidence of significant AHPs in layers 2/3 (Zhao et al., 2016). However, these studies were performed in tissue slices or in vivo using patch‐clamp electrodes and do not necessarily reflect on neuronal Ca^2+^ networks across hundreds of neurons. Thus, here, we focused our attention on S1 and imaged the Ca^2+^ network in layers 2/3, a region that is engaged when encoding proprioception and sensory discrimination.

Each year, 30–40% of individuals over 65 sustain a fall, accounting for the greatest cause of injury‐related morbidity and mortality among older adults. Multiple intervention strategies are recommended to help prevent these falls (Patil et al., 2016; Tricco et al., 2017), yet these are only moderately effective (Lord & Close, 2018). Additionally, the lack of patient adherence and the cost and paucity of these programs significantly reduce their potential impact (Ganz et al., 2015). Recently, rivastigmine was shown to reduce fall frequency by 45% (Henderson et al., 2016). However, this may not be applicable to all individuals, as this study was performed in Parkinson's disease patients, and there are currently few other treatments available that target age‐dependent motor dysfunction, aside from vitamin D supplementation (Patil et al., 2016; Wicherts et al., 2007), further emphasizing the need for investigations into novel therapeutic strategies. Recently, intranasal insulin (INI) has become a recognized therapeutic approach in the clinic, and few negative side effects have been noted in mild cognitively impaired (MCI) or AD patients (Craft et al., 2020). In adult subjects, insulin is detected in the cerebrospinal fluid ~30 min following intranasal (IN) delivery (Born et al., 2002). Interestingly, evidence suggests that not only is INI associated with improvements in cognitive and functional domains (Benedict et al., 2011) but it may also target modalities involved in sensory function and ambulatory control. Indeed, several pre‐clinical and clinical studies have highlighted insulin's ability to promote brain activity in cortical regions, locomotion, and the desire to move (Hennige et al., 2009; Sartorius et al., 2012; Sartorius et al., 2016), supporting its therapeutic potential in the older population. Further, our group has shown that the same neuronal Ca^2+^ processes that are sensitive to aging are also sensitive to insulin (Anderson et al., 2017; Frazier, Ghoweri, Anderson et al., 2019; Maimaiti et al., 2016, 2017; Pancani et al., 2013).

Here, we performed in vivo, two‐photon (2P) Ca^2+^ imaging during tactile activation in anesthetized Fisher 344 (F344) male rats using a Morse continuous wavelet transform (CWT) routine for data extraction and analysis to characterize age‐dependent alterations in the S1 neuronal network and to identify potential cellular components that may underlie these changes. We also measured ambulatory performance in young and aged F344 animals using a 3‐plane visualization walking task to test for links between S1 network communication and locomotor behavior. Compared to young, aged animals had increased overall activity and connectivity of the S1 neuronal Ca^2+^ network along with greater ambulatory variability and slower locomotor speed. In aged animals, acute INI increased network synchronicity, locomotor speed, and motivation to ambulate. Importantly, we also show that young animals receiving the local application of Bay‐K 8644 (an L‐VGCC agonist) to S1 had altered network properties mirroring the phenotype seen in the aged animal. Overall, our work generalizes Ca^2+^ dysregulation to an area of the brain associated with sensory discrimination and ambulation, reinforces the role of L‐VGCCs in altering neuronal network properties in vivo, and provides evidence that insulin may be able to impact ambulatory behavior by altering S1 network communication.

## 2 RESULTS

### 2.1 2P imaging of the neuronal Ca^2+^ network

**FIGURE 1:**
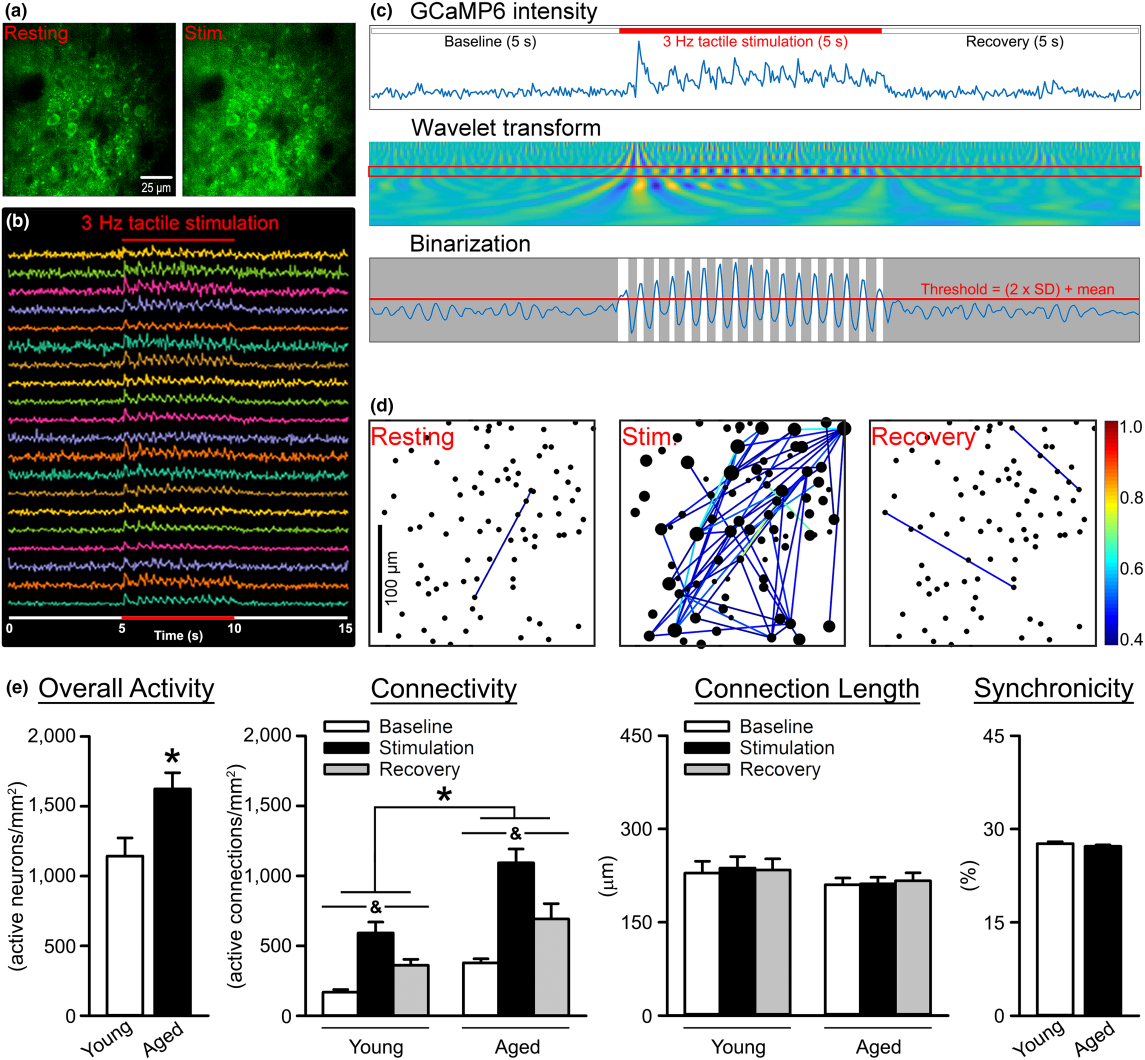
Analysis of the S1 neuronal Ca^2+^ network in young and aged animals. (a) Individual GCaMP6‐positive neurons are distinguishable in S1 before (left) and during (right) tactile stimulation. Dark areas reflect penetrating blood vessels. (b) Representative traces from ROIs in response to tactile stimulation at 3 Hz (red line). (c) (top) Raw GCaMP6 signals before, during (red line), and after tactile stimulation is extracted using a continuous wavelet transform (middle), then thresholded and binarized (bottom). (d) Network connectivity correlogram. The size of the dot (neuron) represents the sum of that neuron's weighted CCs with all other neurons. The color of the line between two dots (see scale bar) indicates the unweighted CC between those neurons. (e) Extracted network properties were obtained in young (*n* = 6 animals, total of 30 FOVs [4685 neurons]) and aged (*n* = 6 animals, total of 51 FOVs [9593 neurons]) F344 rats. A significant aging effect was detected on measures of overall activity (Mann–Whitney Rank Sum Test; *U* = 425.50, *p* < 0.001), with aged animals having a greater number of active neurons/mm^2^ compared to young. As expected, measures of connectivity indicated that pacing of the network via tactile stimulation increased neuronal Ca^2+^ (2‐way RM ANOVA; *F*_[1.89,149.00]_ = 39.89, *p* < 0.0001). A main effect of age was also noted (2‐way RM ANOVA; *F*_[1,79]_ = 13.12, *p* = 0.0005), with aged animals having significantly increased connectivity (active connections/mm^2^) compared to young. No significant changes were detected on measures of connection length or synchronicity (*p* > 0.05). Data represent means ± SEM. Asterisks (*) indicate a main effect of age at *p* < 0.05. Ampersands (&) indicate a main effect of stimulation at *p* < 0.05

Measures of neuronal Ca^2+^ network variables in response to tactile stimulation in young (*n* = 6 animals, total of 30 fields of view [FOVs] [4685 neurons]) and aged (*n* = 6 animals, total of 51 FOVs [9593 neurons]) F344 rats were extracted using a CWT analysis. Results highlighted greater overall activity (Figure 1e; Mann–Whitney Rank Sum Test; *U* = 425.50, *p* < 0.001) along with increased network connectivity (2‐way repeated measure analysis of variance [RM ANOVA]; *F*_[1,79]_ = 13.12, *p* = 0.0005) in aged animals compared to young. As expected, the pacing of the network using tactile stimulation (3 Hz, 5 s) significantly elevated measures of connectivity during the stimulation period (*F*_[1.89,149.00]_ = 39.89, *p* < 0.0001). No significant changes were detected on measures of connection length or synchronicity (*p* > 0.05).

**FIGURE 2:**
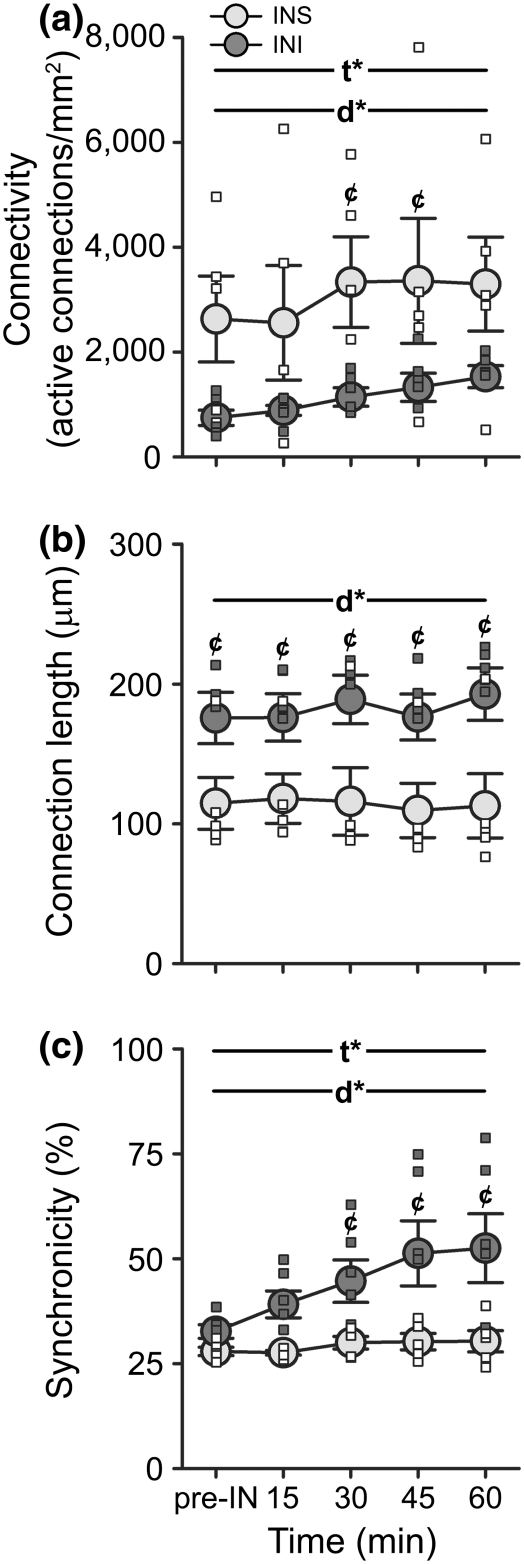
Analysis of the S1 neuronal Ca^2+^ network following INS or INI delivery in aged animals. (a) The main effects of both of drug (2‐way RM ANOVA; *F*_[1,9]_ = 4.99, *p* = 0.05) and time (*F*_[4,36]_ = 2.66, *p* = 0.05) were detected on measures of network connectivity between animals receiving INI (*n* = 4, total of 6 FOVs [911 neurons]) compared to those receiving INS (*n* = 4, total of 5 FOVs [891 neurons]). (b) Similarly, measures of connection length also significantly differed between the two groups (2‐way RM ANOVA; *F*_[1,9]_ = 6.62, *p* = 0.03), with INI‐treated animals displaying longer connections across all timepoints tested (Tukey's post‐hoc test; *p* < 0.05). (c) For measures of synchronicity, the main effects of both time (2‐way ANOVA; *F*_[1.36,12.25]_ = 9.90, *p* = 0.01) and drug (*F*_[1,9]_ = 7.43, *p* = 0.02) were detected, as well as a significant interaction term (*F*_[4,36]_ = 4.20, *p* = 0.01). Data represent means ± SEM. Asterisks indicate the main effects of time (t*) or drug (d*) at *p* < 0.05. Cent symbols (¢) denote Bonferroni post‐hoc significance (*p* < 0.05) between INS and INI at the timepoints indicated

To identify potential processes underlying INI‐mediated improvements in gait, we investigated the S1 network in response to acute IN saline (INS) or INI in a new set of aged animals (INS *n* = 4 animals, a total of 5 FOVs [891 neurons]; INI *n* = 4 animals, total of 6 FOVs [911 neurons]). For measures of connectivity, a main effect of the drug was noted (Figure 2a; 2‐way RM ANOVA; *F*_[1,9]_ = 4.99, *p* = 0.05). A main effect of time was also detected (2‐way RM ANOVA; *F*_[4,36]_ = 2.66, *p* = 0.05), irrespective of the drug delivered. Measures of connection length showed that INI‐treated animals also had greater distances between neurons (Figure 2b; 2‐way RM ANOVA; *F*_[1,9]_ = 6.62, *p* = 0.03) across all timepoints tested (Tukey's post‐hoc; *p* < 0.05). No effect of time was detected on this measure (*p* > 0.05). We also noted an INI‐mediated increase in measures of synchronicity (Figure 2c; 2‐way ANOVA; *F*_[1,9]_ = 7.43, *p* = 0.02) starting 15 min after delivery, as well as a main effect of time (*F*_[1.36,12.25]_ = 9.90, *p* = 0.01). A significant interaction term (*F*_[4,36]_ = 4.20, *p* = 0.01) was also seen on this measure, suggesting that compared to INS, S1 network communication increases over time and is sensitive to insulin.

### 2.2 Impact of Bay‐K 8644 on Ca^2+^ network characteristics

**FIGURE 3:**
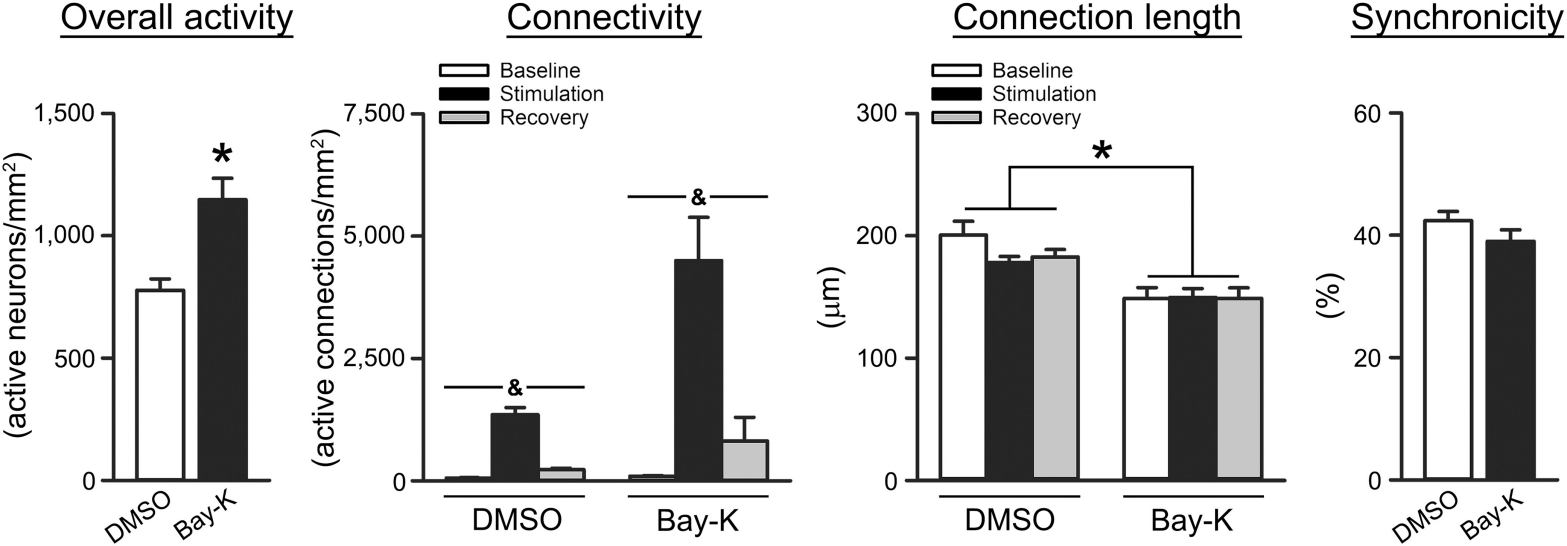
Impact of acute Bay‐K 8644 delivery on the S1 Ca^2+^ network. Extracted network properties obtained in young F344 rats given local S1 application of either 0.1% DMSO (*n* = 2 animals, total of 25 FOVs [2748 neurons]) or 500 nM Bay‐K 8644 (*n* = 2 animals, total of 33 FOVs [3252 neurons]). Bay‐K 8644 (Bay‐K) was associated with increased overall activity compared to DMSO (Mann–Whitney Rank Sum Test; *U* = 281.50, *p* = 0.02). While not significant, a strong trend for an effect of Bay‐K 8644 was noted on measures of connectivity (2‐way RM ANOVA; *F*_[1,56]_ = 2.72, *p* = 0.11). A main effect of stimulation was also detected in this measure (*F*_[1.88,105.10]_ = 281.08, *p* < 0.0001). Measures of connection length indicated that Bay‐K 8644 was associated with shorter distances between neurons (2‐way RM ANOVA; *F*_[1,56]_ = 14.09, *p* = 0.0004). No significant difference in synchronicity was detected between groups (Mann–Whitney Rank Sum Test; *p* > 0.05). All data represent means ± SEM. Asterisks (*) indicate the main effects of the drug at *p* < 0.05. Ampersands (&) indicate a main effect of stimulation at *p* < 0.05

Given robust prior evidence of increases in L‐VGCCs in aged animals (Thibault & Landfield, 1996), we tested whether local delivery of the L‐VGCC agonist Bay‐K 8644 to the S1 of young F344 rats would mimic at least some of the aging phenotype of enhanced network communication reported here (Figure 1). Using this approach, we showed that acute application of 500 nM Bay‐K 8644 (*n* = 2 animals, total of 33 FOVs [3318 neurons]) increased the overall activity of the network (Figure 3; Mann–Whitney Rank Sum Test; *U* = 281.50 *p* = 0.02) compared to 0.01% dimethyl sulfoxide (DMSO) (*n* = 2 animals, total of 25 FOVs [2807 neurons]). Measures of connectivity were not significantly different between treatment groups, although a strong trend was detected (2‐way RM ANOVA; *F*_[1,56]_ = 2.72, *p* = 0.11). Delivery of Bay‐K 8644 also significantly reduced measures of connection length (2‐way RM ANOVA; *F*_[1,56]_ = 14.09, *p* = 0.0004). No significant difference in synchronicity was detected between groups (Mann–Whitney Rank Sum Test; *p* > 0.05).

### 2.3 Locomotor alterations with age

**FIGURE 4:**
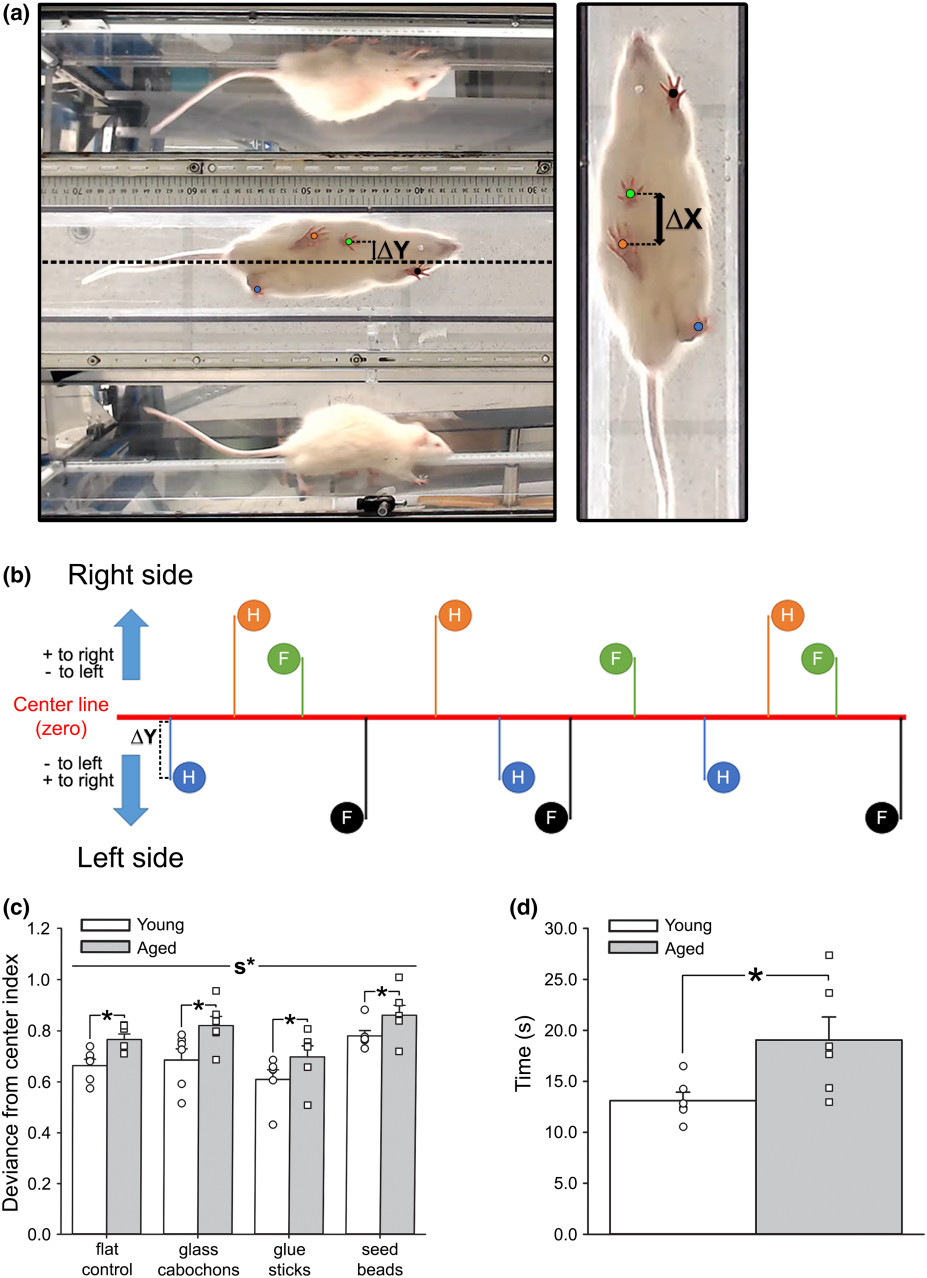
Impact of age on measures of ambulatory performance. (a) Example of a frame taken from a gait recording showing the view from underneath the clear, flat surface corridor, as well as the mirror reflections of the side views. Centerline, Δ*X*, and Δ*Y* are marked. (b) Representation of the calculation of deviance from the center. The Δ*Y* from the center line of each hindpaw (H) and forepaw (*F*) placement was summed into one value. This value was then normalized to the number of steps and the corridor width to derive deviance from the center index. (c) Measures of deviance from the center index obtained in young (*n* = 6) and aged (*n* = 6) F344 animals showed a significant difference between surfaces (2‐way RM ANOVA; *F*_[2.23,22.29]_ = 10.84, *p* = 0.0004), as well as a significant impact of age (2‐way RM ANOVA; *F*_[1,10]_ = 9.53, *p* = 0.01), with young animals having improved gait performance compared to aged across all surfaces tested. (d) Aged animals took significantly longer to complete the four trials (one per each surface) compared to young (Student's *t* test; *p* = 0.03), indicating an age‐related gait impairment. All data represent means ± SEM. The main effect of the surface is indicated by s*, while asterisks (*) represent the main effects of age

**FIGURE 5:**
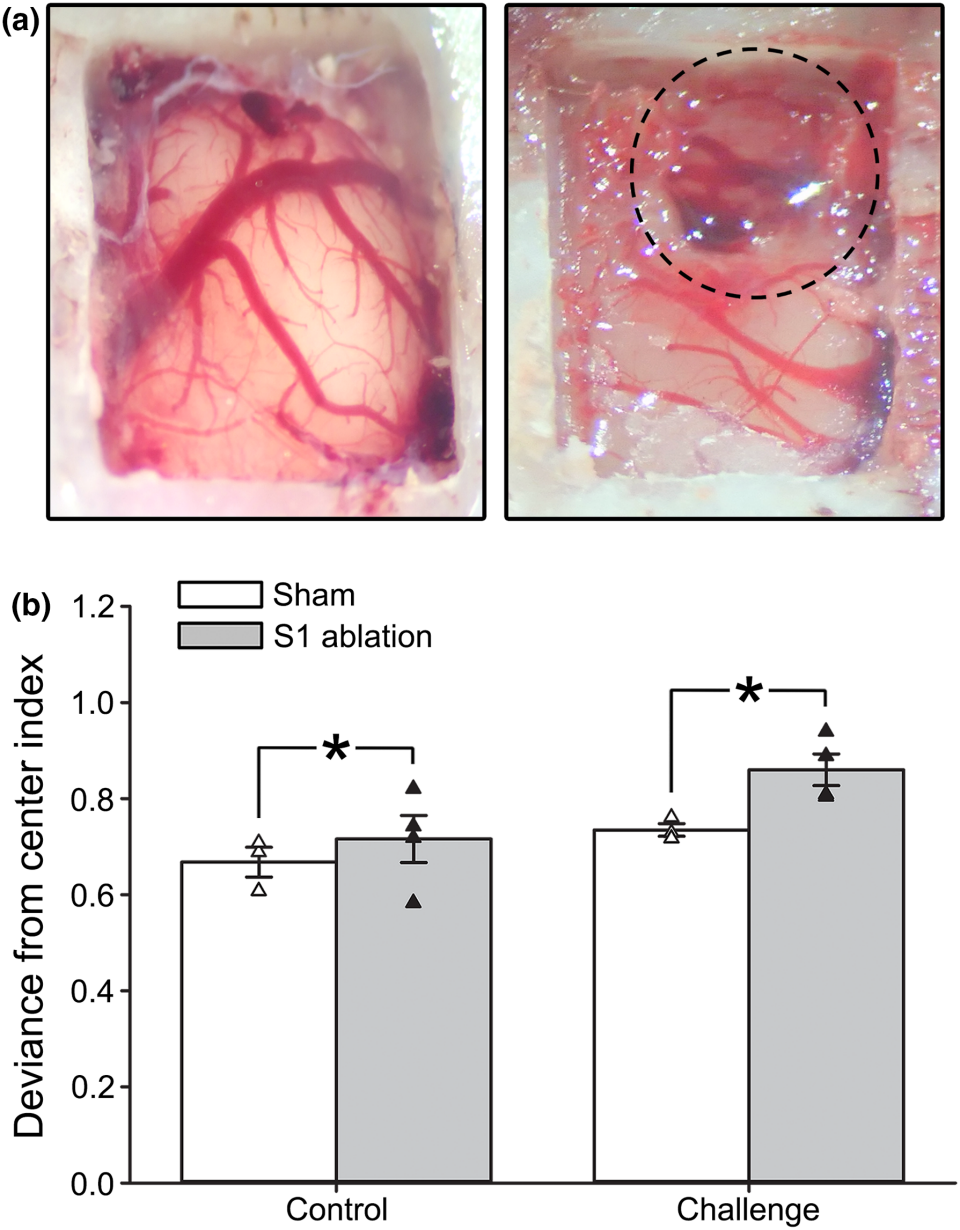
Impact of S1 ablation on measures of ambulatory performance in young and aged animals. (a) Images of craniotomies performed in F344 rats following sham (left) and S1 ablation (right) procedures. The dotted line represents the location of the S1 ablation. (b) Measures of deviance from the center index were obtained in a subset of animals subjected to either a sham (*n* = 3 [2 young, 1 aged]) or S1 ablation (*n* = 4 [2 young, 2 aged]). Animals were tested on two different surfaces (flat control and seed bead challenge). Results indicate that S1 ablation was associated with greater deviance from center index scores compared to sham across both of the surfaces tested (2‐way RM ANOVA; *F*_[1,5]_ = 9.94, *p* = 0.03). All data represent means ± SEM. Asterisks (*) indicate significance at *p* < 0.05

We investigated ambulatory performance in young (*n* = 6) and aged (*n* = 6) F344 rats across four distinct surfaces using a 3‐plane visualization walking task (Figure 4). A main effect of age was identified (Figure 4; 2‐way RM ANOVA; *F*_[1,10]_ = 9.53, *p* = 0.01), with aged animals displaying increased paw position variability during ambulation. We also detected a main effect of surface (*F*_[2.23,22.29]_ = 10.84, *p* = 0.0004), highlighted by increased deviance from the center index on the seed bead surface compared to flat control (Šidák's multiple comparisons post‐hoc test; *p* < 0.05) or glue sticks (*p* < 0.0001). As expected, aged animals took significantly longer to complete the task (Figure 4d; Student's *t* test; *p* = 0.03). Other parameters tested included indices associated with coordination, paw‐precision, total stride deviance, and paw crossover rates, but no aging differences were noted (*p* > 0.05; *data not shown*). To test if S1 activity impacts gait performance as part of the sensorimotor loop engaged during ambulation, S1 ablations were performed in a subset of animals (sham *n* = 3 [2 young, 1 aged]; S1 ablation *n* = 4 [2 young, 2 aged]) followed by gait measures across two surfaces (flat control and seed beads). S1 ablation resulted in greater deviance from center index scores, particularly on the more challenging seed bead surface (Figure 5; 2‐way RM ANOVA; *F*_[1,5]_ = 9.94 *p* = 0.03), providing some evidence that S1 participates in the neuronal circuit engaged during ambulation. Overall, our results indicate that we can reliably detect alterations in certain characteristics of locomotor performance in both young and aged animals.

### 2.4 Effect of acute INI on locomotor stability

**FIGURE 6:**
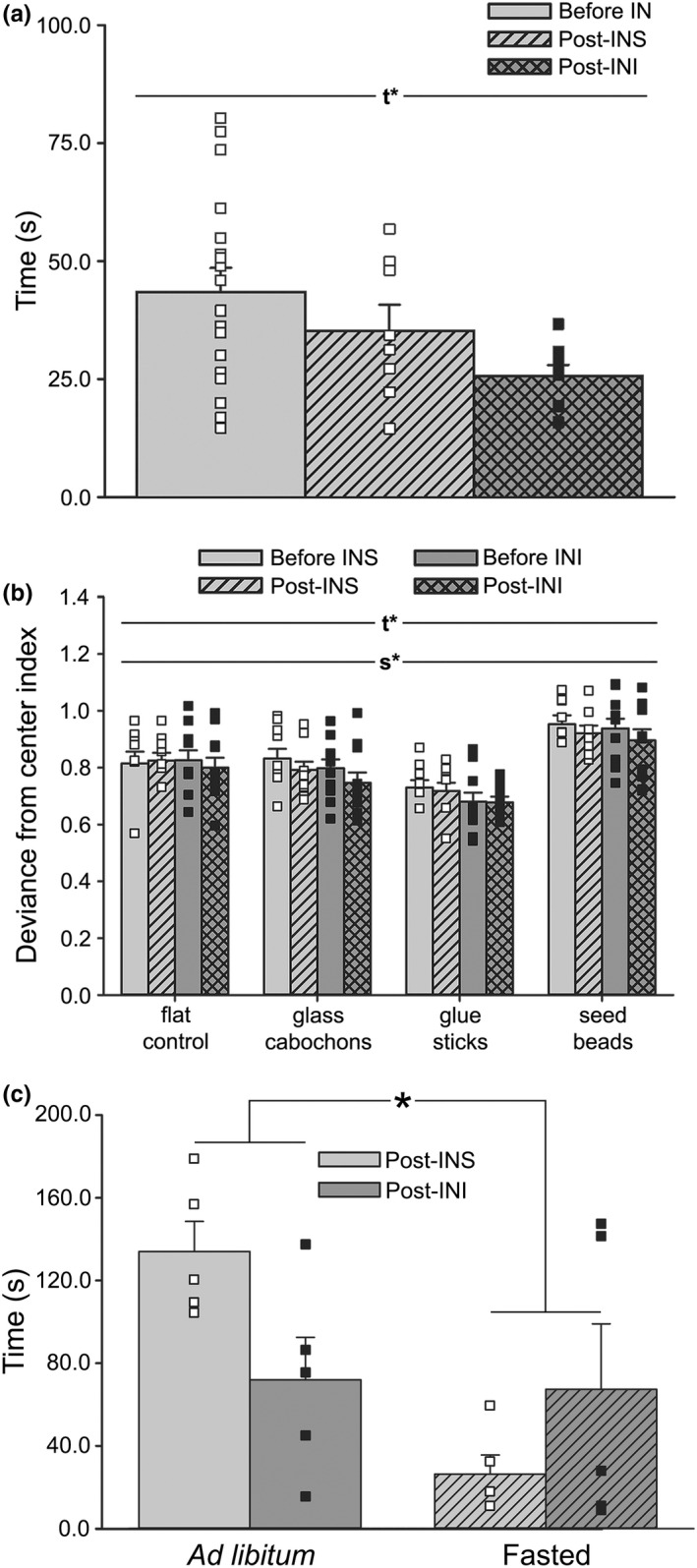
Impact of acute INI administration and motivation on measures of ambulatory performance in aged animals. (a) Measures of time taken to ambulate down the corridor on 4 different surfaces were obtained in aged animals (*n* = 18) treated with INS (*n* = 8) or INI (*n* = 10). Results indicate that INI significantly reduced the time needed to complete the four trials compared to INS (Welch's ANOVA; *W*_[2,16.23]_ = 6.40, *p* < 0.01). (b) Deviance from center index scores was significantly different by both time (before vs. post‐treatment; 3‐way RM ANOVA; *F*_[1,16]_ = 6.56, *p* = 0.02) and surface (3‐way RM ANOVA; *F*_[2.41,38.52]_ = 52.93, *p* < 0.0001), with seed bead showing greater deviance measures compared to all other surfaces (Šidák's multiple comparison post‐hoc test; seed bead vs. flat control *p* < 0.001, vs. glass cabochon *p* < 0.0001, vs. glue sticks *p* < 0.0001), indicating that seed beads were the most challenging surface. All data represent means ± SEM. Main effects of time (t*) or surface (s*) at *p* < 0.05 are indicated. (c) Average time spent in the corridor was obtained in aged animals receiving INS (*n* = 5) or INI (*n* = 5) under ad libitum or fasted feeding conditions. Results indicated that time spent in the corridor was significantly reduced under fasted conditions (2‐way ANOVA; *F*_[1,16]_ = 7.34, *p* = 0.02). Additionally, a significant interaction term was also detected (*F*_[1,16]_ = 6.19, *p* = 0.02), with INI‐treated animals ambulating more quickly than INS‐treated under ad libitum conditions, while this effect was reversed when animals fasted. Data represent means ± SEM. Asterisk (*) indicates significance at *p* < 0.05

Next, we tested the impact of acute INI on ambulatory behavior. These measures were conducted only in aged animals, as our prior work (Anderson et al., 2017; Frazier, Ghoweri, Sudkamp et al., 2019; Maimaiti et al., 2016) has shown little, if any, INI effects in young animals. The ambulatory performance of all animals (before IN, *n* = 18) was initially evaluated prior to IN delivery treatment. Animals were then split into two groups and treated with either INS (*n* = 8) or INI (*n* = 10). One hour later, the animals were tested for changes in locomotion a second time. Comparison of performance between the two groups prior to INS or INI treatment showed no difference (Student's *t* test; *p* = 0.77) and were thus combined (before IN) prior to statistical comparison. Analysis of time revealed a main effect of treatment, with post‐INI animals completing the task more quickly across all surfaces (Figure 6a; Welch's ANOVA; *W*_[2,16.23]_ = 6.40, *p* < 0.01). While INI did not specifically alter gait performance based on analysis of deviance from the center, a main effect of the surface was noted (Figure 6b; 3‐way RM ANOVA; *F*_[2.41,38.52]_ = 52.93, *p* < 0.0001), highlighted by increased deviance from center index scores on the seed bead surface compared to all others (Šidák's Multiple Comparison post‐hoc test; seed beads vs. flat control *p* < 0.001; vs. glass cabochon *p* < 0.0001; vs. glue sticks *p* < 0.0001).

### 2.5 Interactions between motivation and acute INI on ambulatory performance

To determine if INI could impact the motivation to ambulate, another set of 10 aged animals were evaluated following either a time‐restricted (fasted) or unrestricted (ad libitum) feeding paradigm. The end of the corridor was baited with Froot Loops^®^ cereal to motivate the animals. On the day of testing, animals received an acute dose of INS (*n* = 5) or INI (*n* = 5) ~60 min prior to ambulating. Analysis of time revealed a main effect of the feeding paradigm, with fasted animals ambulating more quickly (Figure 6c; 2‐way ANOVA; *F*_[1,16]_ = 7.34, *p* = 0.02), likely mediated by motivation from the food reward. Importantly, INI‐treated animals ambulated faster than INS‐treated under ad libitum conditions, and this effect was reversed when animals fasted, as highlighted by a significant interaction term (*F*_[1,16]_ = 6.19, *p* = 0.02). Given that non‐motivated animals (ad libitum) responded to INI with an increase in speed while motivated animals (fasted) did not, these results suggest some level of interaction between ambulatory motivation and INI, as seen previously (Hennige et al., 2009; Sartorius et al., 2012; Sartorius et al., 2016).

## 3 DISCUSSION

This study examined the presence of Ca^2+^ dysregulation and its impact on neuronal network communication in S1, a region that has received little attention in the field of aging compared to the hippocampus. Variables included measures of neuronal activity, connectivity, connection lengths, and synchronicity. We also identified two elements underlying this Ca^2+^ dysregulation in the network: L‐VGCCs and insulin receptors. Additionally, this work also investigated the role of Ca^2+^ dysregulation not only on sensory information but also on gait control.

Results from measures of efficiency in population coding and neuronal synchronization in aged primates (Overton & Recanzone, 2016) and humans (Bauer et al., 2006) indicate that central processing associated with attention in the visual field and sensory discrimination in S1 reflect the strength of the network. In S1, this encoding represents vibration, tactile sensations, limb position, and 2‐point discrimination (Prsa et al., 2019). Similarly, population‐level network performance is critical for learning and is echoed across different areas of the brain, including the auditory, visual, and motor cortex, and decreased performance in these regions is seen with aging (Alexander et al., 2020;Hickmott & Dinse, 2013; Thome et al., 2016). Pioneer work using electrophysiological approaches to infer firing rates across tens of neurons in the aged brain describes reduced place‐field selectivity and decreased reliability of environmental mapping and encoding in the hippocampus of aged rats (Barnes et al., 1983; Mizumori et al., 1996; Tanila et al., 1997). While S1 slice recordings from aged rats showed increased responses and cellular excitability during trains of thalamocortical activation compared to adult animals (Hickmott & Dinse, 2013; Popescu et al., 2021), this work used patch‐clamp techniques and was limited by the number of neurons characterized. Further, only a handful of imaging studies have directly characterized neuronal Ca^2+^ dynamics in field CA1 of the aged hippocampus ex vivo [reviewed in (Frazier et al., 2017)]. Here, our results show alterations in the S1 network and identify significant increases in measures of overall activity and connectivity (Figure 1e), suggesting that based on these network measures, Ca^2+^ dysregulation with aging may be generalizable to areas outside the hippocampus. This is consistent with prior investigations of population encoding that showed age‐dependent increases in synaptic activation and excitability in S1 and temporal structures using electrophysiology (Hickmott & Dinse, 2013; Thome et al., 2016) and throughout the brain using magnetic resonance imaging (Alexander et al., 2020). Work in the clinic has reported significant increases in S1 excitability concomitant with impaired tactile acuity in aged individuals (Lenz et al., 2012) along with altered cortical‐proprioceptive processing with aging (Piitulainen et al., 2018) and increased beta band suppression in response to a proprioceptive stimulus that is correlated with decreased sensorimotor function (Bardouille et al., 2019; Walker et al., 2020). Evidence in animal models support these findings of increased neuronal S1 excitability with aging that is perhaps mediated by altered GABAergic innervation (Hickmott & Dinse, 2013; Popescu et al., 2021; Spengler et al., 1995). In contrast, other work has shown age‐dependent decreases in functional connectivity in older, cognitively impaired, anesthetized rats (Ash et al., 2016), while another group reported that although electrophysiological measures in young and old mice showed a decrease in gamma and theta activity with age, overall neuronal excitability remained unchanged (Jessen et al., 2017). Here, our results showed no change in connection lengths between pairs of neurons reported (Figure 1e), suggesting similar or common inputs onto the network were likely activated by the stimulation and implying that the size of the network did not change with age. However, note that we could not evaluate field size in the current study due to the restrictive size of our FOV (~180 × 180 μm), nor the distribution of responses across multiple frequencies during baseline conditions (without stimulation) due to the short 5 s baseline period used for these experiments.

Prior work in the field of aging has highlighted the importance of neuronal Ca^2+^ dysregulation within single cells and across selected brain regions (i.e., the forebrain, cerebellum, cortex, and hippocampus) (Gant et al., 2006; Kumar & Foster, 2005; Moyer Jr et al., 1992; Norris et al., 1998; Oh et al., 2016; Stutzmann et al., 2006; Thibault et al., 2001), yet very little work has investigated this process across a multitude of activated cells within a functioning network. While changes in basal firing rates reflect learning (Weible et al., 2006) and reduced rates have been attributed to enhanced Ca^2+^‐dependent AHPs (McEchron et al., 2001), correlations between these variables represent different sources, including trans‐synaptic, as well as post‐synaptic, functions. As the measures of Ca^2+^ changes presented here are synaptically driven, they provide insights into neuronal network dynamics that may be independent of the post‐synaptic AHP. Two prior studies investigating changes in neuronal excitability with age showed increased excitability in cerebral networks (V1 and S1), yet did not identify changes in the AHP (Hickmott & Dinse, 2013; Popescu et al., 2021). A third study also recorded neurons of S1 layers 2/3 and layer 5 in vivo did not identify significant AHPs in layers 2/3 (Zhao et al., 2016). Thus, it is difficult for us to comment on the links between Ca^2+^ dysregulation, traditional measures of the AHP, and ambulatory performance without further electrophysiological characterization of layer 2/3 neurons with aging. Furthermore, this prior work was performed at the single‐cell and tetrode level and does not necessarily reflect on the performance of a network that is engaging hundreds of neurons during a task, and we are unaware of any reports investigating neuronal Ca^2+^ networks across hundreds of neurons using in vivo Ca^2+^ imaging approaches in the aged animal. Thus, using 2P imaging, we attempted to identify potential Ca^2+^‐mediated mechanisms underlying alterations in the network (Figure 1e), and also tested for the contribution of L‐VGCCs, as they are well‐characterized, robust biomarker of aging (Moyer Jr et al., 1992; Norris et al., 1998; Oh et al., 2016; Thibault & Landfield, 1996). Indeed, the use of L‐VGCC blockers has been associated with improved gait in aged rats (Van der Zee et al., 1990), highlighting the association between neuronal Ca^2+^ and ambulatory behavior. While the mechanisms responsible for hyperactivity in S1 in the aged animal remain largely unknown, here, local application of the L‐VGCC agonist Bay‐K 8644 to the S1 of young rats was able to partially reflect some aspects of the aging phenotype (Figure 3). Specifically, evidence of Bay‐K 8644‐mediated increases in overall activity and connectivity in young animals suggests a potential mechanism underlying the enhancement of network activity seen across hundreds of neurons in aged animals, and perhaps the alterations in ambulatory behavior (Figure 4).

In the clinic, the ambulatory function is assessed via measures of locomotion such as stride, speed, and deviance from the center (Tian et al., 2017). Increased gait variability is associated with a higher risk of falls in normal aging, MCI, AD, and dementia (Pieruccini‐Faria et al., 2021), and has also been correlated with structural/ functional changes in S1, the hippocampus, anterior cingulate gyrus, and parts of the basal ganglia (Beauchet et al., 2019; Bolandzadeh et al., 2014; Clark et al., 2019), where it appears to be an excellent predictor of future cognitive dysregulation. In the current study, we characterized different aspects of ambulatory behavior that are also sensitive to aging using a 3‐plane visualization walking task (Figure 4). Our findings align well with clinical assessments, as others have also highlighted similar age‐related alterations in the F344 rat. Indeed, one study reported worsened locomotor performance and function in aging despite the absence of sarcopenia (Horner et al., 2011), while another highlighted impaired walking behavior, increased receptive field sizes, and slower responses to stimulation (David‐Jurgens et al., 2008). In the latter study, these changes coincided with altered S1 neural processing and cortical mapping, suggesting a brain‐centric mechanism may be involved. Indeed, while peripheral dysregulation plays a significant role in age‐related ambulatory distress, evidence of central dysregulation has also been reported across brain areas, including network dysfunction during encoding (Thome et al., 2016), and, in AD patients, greater S1 size associated with larger gait variability (Beauchet et al., 2019). These findings support the need for therapeutic approaches with a focus on central processes.

Most prior work using INI as a clinical therapy has focused on its ability to enhance cognitive performance in diabetic and AD patients or its function in young, healthy adults [reviewed in (Freiherr et al., 2013)]. However, INI has also been shown to increase the desire to move in both clinical and pre‐clinical settings (Hennige et al., 2009; Sartorius et al., 2012; Sartorius et al., 2016), and previous results from our lab using a constitutively active insulin receptor reported reduced stride length in both young and aged F344 animals (Frazier et al., 2020); thus, it is not surprising that insulin also mediated aspects of gait performance here (Figure 6). Interestingly, our results appear to depend on the motivational state, as ad libitum‐fed aged animals treated with INI had decreased ambulatory times while fasted animals did not (Figure 6c), suggesting insulin may impact reward/ motivational pathways. However, as our work does not show that age‐associated Ca^2+^ changes (i.e., overall activity and connectivity) in S1 are offset by INI, future studies are needed to determine whether these changes in ambulatory behavior are reflective of increased Ca^2+^ network synchronicity (Figure 2).

Our data cannot address whether the results reported here manifest because of an age‐dependent sensitivity to anesthesia. However, while behavioral states likely alter network performance, this does not necessarily obviate before/after comparisons in experiments using acute INI or L‐VGCC modifiers for mechanistic investigations. Further, little to no change in the mean frequency of Ca^2+^ transients and the number of hyperactive neurons in the hippocampal CA1 field has been detected between awake and anesthetized mice using in vivo 2P Ca^2+^ imaging (Yao et al., 2022). While amplitude differences in cortical hemodynamic imaging and neurovascular unit (NVU) activation (i.e., oxyhemoglobin and deoxyhemoglobin) and increases in network synchronicity are seen using anesthesia, the direction of change in response to whisker stimulation is unaffected (Martin et al., 2013). Similarly, based on visual field data, although network correlations between neurons increase with anesthesia, orientation selectivity does not change, suggesting population‐wide alterations in coding are not always present (Goltstein et al., 2015). Furthermore, preliminary data from our lab showed very similar changes in Ca^2+^ network responses during tactile stimulation between anesthetized and awake, head‐restrained mice during ambulation across a coarse surface (*data not shown*).

Here, we used a network analysis strategy to extract power and frequencies in complex signals (Lilly, 2017) and characterize in vivo neuronal Ca^2+^ kinetics. We provide some of the first evidence that Ca^2+^ dysregulation is present outside of the hippocampus in vivo in the aged F344 rat and that the Ca^2+^ network variables measured here are sensitive to L‐VGCC modifiers. Moreover, it is satisfying to note that this approach appears valid, as the high‐power frequency domain extracted aligns well with the 3 Hz tactile stimulation frequency (Figure 1c). Our work supports the need for further investigations of L‐VGCCs as potential therapeutic targets for gait disorders with aging. We also present evidence that INI can alter network synchronicity in S1 and may offset some aspects of gait dysfunction with age, perhaps through increases in motivation. Additionally, we suggest that modalities other than those associated with cognitive function and the hippocampus are sensitive to INI and should be further considered as therapeutic targets in the clinic.

## 4 EXPERIMENTAL PROCEDURES

### 4.1 AAV delivery

The work presented here strictly adheres to our Institutional Animal Care and Use Committee protocol. Approximately 4–6 weeks prior to 2P imaging, young (*n* = 10) and aged (*n* = 14) male F344 animals received aseptic injections of AAVs (1–3 e13 GC/ml) carrying the neuron‐specific Ca^2+^ indicator GCaMP6 (AAV.CamKII.GCaMP6s.WPRE.SV40; Addgene #107790). Briefly, animals were anesthetized (1.5–2.5% isoflurane) and placed on a heated pad (37°C). Artificial tears (GenTeal^®^) were placed on each eye and 2–3 ml warm saline was administered subcutaneously. PhysioSuite (Kent Scientific Corporation, Torrington, CT) was used to monitor vitals (heart and respiratory rate, body temperature, and O_2_ saturation) and control the heated pad. Burr holes (0.5 mm diameter) were then drilled stereotaxically (Neurostar Drill and Injection Robot with automatic depth detection, Tubingen, Germany). The advancement and retraction speed of the drill was 1.0 mm/min. AAV injection was accomplished (ML = ± 2.25 mm; AP = −1.10 mm; DV = −0.40 mm) using a Hamilton^®^ syringe with a 30° bevel (2.0 μl/side, 0.2 μl/min). Burr holes were then sealed using a bonding agent (VivaPen^®^, Ivoclar Vivadent, Schaan, Liechtenstein) and light‐curing dental cement (Fusion Flo, Prevest Denpro Ltd., Jammu, India). Subcuticular sutures were used to close the wound, followed by a single injection of meloxicam (2.0 mg/kg) and buprenorphine (0.02 mg/kg). Animals were temporarily placed in a heated cage for recovery until fully awake, then returned to their home cage. Post‐operative pain management was controlled by daily application of subcutaneous buprenorphine for 2 days. Animals underwent a 4–6 week recovery period, at which point 2P imaging was initiated.

### 4.2 Acute terminal craniotomies for 2P imaging

On the day of imaging, animals were prepped with the construction of an acute cranial window, followed immediately by 2P imaging. Briefly, animals were anesthetized with urethane‐based anesthesia supplemented with alpha‐chloralose (500 mg/kg urethane, 120 mg/kg alpha‐chloralose in H_2_O) using IP injection and placed on a heated pad for the remainder of the day. The skin on top of the skull was removed to expose the bone ridges and the skull was partially flattened using a hand drill (Foredom, Bethel, CT). After abrasion of the bone surface, a craniotomy (4 × 5 mm) was accomplished using the Neurostar drill, followed by a durotomy (Figure 5a). The space above the brain was irrigated with cold saline, then quickly filled with 1.5% low‐temperature agar in saline. A glass coverslip (5 × 6 mm) and carbon‐fiber head plate were attached to the bone using a bonding agent and dental cement. The animal was subcutaneously injected with lactated Ringer's solution (2–3 ml) and then immediately transported to the 2P microscope for imaging. Supplemental doses of anesthesia were provided as needed at 1/10th of the initial dose.

### 4.3 Tactile stimulation and 2P imaging

Imaging during ambulatory movement would be ideal to investigate gait‐related functions; however, this work is challenging in rats compared to mice, particularly in older, larger animals, as their greater strength could compromise the integrity of the head restraint. Few studies have imaged the Ca^2+^ network in rats during awake head‐fixed imaging, and only one has imaged the network during ambulation using a head‐mounted 2P microscope while imaging the visual cortex (Sawinski et al., 2009). Thus, here, we used tactile stimulation to measure S1 activation in anesthetized animals. Electrical leads were placed under the skin of the animal's fore‐ and hindpaw at a depth of ~3 mm. A ground lead was placed medially on the same limb. We chose to activate the network at 3 Hz as this is a physiologically relevant frequency with regard to motor function (Huttunen et al., 2008). A Grass Instruments SD9 stimulator (Astro‐Med Inc., West Warwick, RI) was used to deliver a stimulation (5 s, 10 ms pulse duration) of 10 V, as this was the minimum voltage necessary to reliably trigger subtle digit movements in all animals tested. 2P imaging was accomplished in layers 2/3 of S1 (250–400 μm below the dura) using a Scientifica Hyperscope (Uckfield, United Kingdom) controlled by ScanImage (v2021.0.0; Vidrio Technologies, Leesburg, VA) under MATLAB (vR2020b; MathWorks, Natick, MA) that was equipped with scanning mirrors (1 resonant, 4 galvo) and a large back aperture objective for deep imaging (16×, NA = 0.8; WD = 3.0 mm; Nikon, Tokyo, Japan). FOVs were randomly selected and, if responsive, were stored for further analysis. When multiple FOVs were identified in the same animal, care was taken to ensure no overlap occurred between FOVs. Data were acquired with ScanImage using two GaAsP detectors mounted inside a multiphoton detection chamber (MDUXL) that houses dichroic and infrared blocking filters. GCaMP6 was excited at 930 nm using an InSight X3 dual wavelengths femtosecond‐pulsed laser (Spectra‐Physics, Milpitas, CA). An acquisition computer was configured to digitize data, drive microscope components, and control TTL pulses for syncing with extraneous stimulations. We acquired full frames (512 × 512 pixels) at a rate of 30 Hz before, during, and after (5 s each) tactile stimulation.

For characterization of the neuronal Ca^2+^ network across age in response to tactile stimulation, we performed 2P imaging on 12 animals (Figure 1; young *n* = 6, aged *n* = 6). For characterization of the network in response to INS or INI, another set of 8 aged animals (Figure 2; *n* = 4 per group) were first anesthetized and imaged prior to undergoing IN administration (pre‐IN). Animals were then removed from the stage, positioned supine, and given two 5 μl boluses (1 min apart) of INS or INI to the right naris following our standard protocol. Animals were then placed back on the stage and the same FOV was imaged again every 15 min for 1 h. Because of the repeated nature and time constraints associated with this experiment, fewer FOVs were imaged. To investigate potential Ca^2+^ sources responsible for changes in the network activity, we tested the impact of acute Bay‐K 8644 (an L‐VGCC agonist) administration on neuronal Ca^2+^ dynamics in 4 young animals (Figure 3). Briefly, S1 was exposed to 12 μl of either vehicle (0.1% DMSO; *n* = 2 animals, total of 25 FOVs [2748 neurons]) or 500 nM Bay‐K 8644 (*n* = 2 animals, total of 33 FOVs [3252 neurons]) delivered in artificial cerebrospinal fluid (Pancani et al., 2013) prior to closing the craniotomy. Animals were then imaged on our 2P microscope ~1 h later (see Section 4.3).

### 4.4 Image processing for analysis of Ca^2+^ transients

Signal processing, data extraction, and network visualization of imaging data were accomplished using a custom MATLAB pipeline. Image stacks of each file were imported as cubes (X and Y [pixels] and timepoints). Regions of interest (ROIs) of individual neurons were first determined by adaptive thresholding (sensitivity at 0.52) of the image (all timepoints averaged), then by filtering using a 60–600 pixel limit to remove any undersized or oversized ROIs, as these likely represent imaging artifacts or non‐neuronal cells. All FOVs were checked for the presence of clear, morphologically distinct single neurons (i.e., donuts) to be included in our analysis. The thresholded and filtered image was then used to extract raw GCaMP6 signal intensities across time (traces) for all ROIs in the FOV (Figure 1b). For each ROI, the raw trace (Figure 1c, top) underwent a Morse CWT (Figure 1c, middle; (Lilly, 2017)), allowing us to measure the power across frequencies (0.06–13 Hz). The peak power within the 3 ± 0.5 Hz window of the CWT spectrum (Figure 1c, middle; red box) was then determined for each trace, and response intensities were thresholded and binarized (Figure 1c, bottom). An event was identified if the power was >2 times the standard deviation from the mean of the baseline prior to stimulation, providing a dataset of 1 s (events) and 0 s (non‐events).

### 4.5 Extraction of neural network outcome variables

Binarized events were used to calculate a correlation coefficient (CC) for each pair of neurons across time (Figure 1c, top; baseline, stimulation, and recovery; 5 s each). We initially selected a thresholded CC value of >0.4, allowing us to select for cells with coinciding activity 40% of the time. We then focused on only active connections (periods of time when cells fire together) by defining a weight factor (WF), which was calculated as the ratio of all detected events divided by the total number of samples (30 Hz × 15 s = 450 samples per neuron; total of 900 samples for each pair). A weighted CC was derived by multiplying each pair's initial CC by their WF. Overall activity (active neurons/mm^2^) was calculated by dividing the number of active neurons with at least 1 event during tactile stimulation in each FOV by the FOV's area. For measures of S1 network connectivity (sum of active connections per mm^2^) and connection lengths (average distance [in mm] between each pair along the *X*, *Y* plane), we limited our analyses to only pairs with a weighted CC value >0.02 and only considered FOVs with >100 active connections per mm^2^ during stimulation and at least 1 active connection during baseline. Network synchronicity (%) was calculated by dividing the number of consecutive events detected during 5 s stimulation by the maximum number of events triggered during that period (3 Hz × 5 s = 15 events) and is provided as a percentage of the time.

### 4.6 Gait measures

For comparison of gait across age, young (4 months; *n* = 8) and aged (18 months; *n* = 10) male F344 rats were recorded (HD camera, 1280x720, 50 Hz frame rate) ambulating across our 3‐plane visualization walking task (Figure 4a). Video files were analyzed in ImageJ (v1.52S) using standard *x*‐ and *y*‐axes pixels measures. Of the 18 animals tested, 14 (young *n* = 6, aged *n* = 8) had previously received GCaMP6 adenovirus (AAV) injections for measures of the S1 Ca^2+^ network (*see AAV Injections*), while the remaining 4 (young *n* = 2, aged *n* = 2) received mechanical S1 ablations using gentle abrasion of the cortex during the craniotomy (Figure 5a). After 4 weeks of recovery, animals were acclimated to the task by walking down the flat control surface for multiple training days (3 days, 4 trials/day). Following acclimation, animals were recorded ambulating across 4 surfaces (flat control, glass cabochons, glue sticks, and seed beads). Because of differences in haunch sizes between young and aged animals, two corridor widths were used (young corridor width = 8.2 cm and aged corridor width = 9.2 cm) to normalize measures. Animals that stopped midway through the task were placed at the beginning of the corridor and re‐tested until they completed at least 4 continuous walking steps per surface. One aged rat within the GCaMP6 group died following AAV injection. Another aged animal was unable to perform the task and was removed from the analysis. We report gait measures across the age from 12 GCaMP6‐treated animals (Figure 4; young *n* = 6, aged *n* = 6). Ablated animals were analyzed separately by comparing them to a subset of GCaMP6‐treated sham animals (young *n* = 2, aged *n* = 1) that underwent AAV injection the same week ablations were performed (Figure 5b).

For comparison of gait in response to INI, a new set of 24 aged animals was evaluated. Animals were acclimated to the walking task by allowing each animal to ambulate down the corridor a total of 3–6 times across 1–2 days of training. Following acclimation, animals were recorded walking down each surface until at least 4 continuous steps were recorded. Animals then received INS or INI (insulin aspart; Novo Nordisk Inc., Plainsboro, NJ) using our standard IN protocol (Anderson et al., 2017; Frazier et al., 2020; Maimaiti et al., 2016). We chose a concentration of 0.0715 IU/10 μl insulin aspart, as this is equivalent to the effective dose used in some clinical trials and has been shown to improve age‐related cognitive deficits and alter gene expression profiles in the hippocampus (Frazier, Ghoweri, Sudkamp et al., 2019; Maimaiti et al., 2016). Investigators were blind to treatment groups. Approximately 1 h after IN delivery, animals were recorded walking across all surfaces a second time. For analysis of time, we combined all animals into a single pre‐treatment group (Figure 6a; “before IN”). One animal was removed from the analysis due to respiratory distress. Five others were removed for not reaching the criterion of finishing the task in under 100 s (*see* Section 4.7). Of these animals, 4 did not reach the criterion prior to IN delivery (pre‐IN). One animal was removed for refusing to ambulate (>380 s) following INS exposure. This animal was identified as an outlier (Grubb's test; *Z* = 2.648, *p* < 0.05) and was removed from the analysis. We present gait measures in response to INS or INI from 18‐aged animals (INS *n* = 8, INI *n* = 10).

To characterize the impact of INI on ambulatory motivation, another set of aged animals (*n* = 10) was evaluated (Figure 6c). As the seed bead surface appeared to be the most challenging, these animals were only tested on this surface (2 trials/day). For 2 weeks prior to testing, animals were exposed to 2–3 pieces of Froot Loops^®^ Breakfast Cereal daily to acclimate them to the novel reward. To motivate the animals to perform, we utilized a time‐restricted feeding protocol. Briefly, animals were food deprived for ~15 h prior to ambulating, then given food ad libitum following completion of the task. On day 1, all animals received INS ~1 h prior to performing the task and the corridor was baited with Froot Loops^®^ at both the middle and end. On days 2–3, half of the animals continued to receive INS (*n* = 5) while the other received INI (*n* = 5), and only the corridor end was baited. Two weeks later, animals were tested again with unrestricted feeding to evaluate the impact of the motivational state on performance. Treatment was switched during this second phase of testing, with the initial INS group receiving INI and vice versa. Unlike our investigations of gait across age and INI, no training or criterion for a number of continuous steps was imposed for these measures.

### 4.7 Quantification of ambulatory performance

Time (s) spent ambulating (time between corridor entry and the moment the animal reached the exit) was summed across all 4 surfaces to calculate each animal's total time. For investigations of gait across age (Figure 4) or in response to INI (Figure 6), animals that took >100 s to ambulate across all surfaces were removed from the analysis. For investigations of ambulatory motivation (Figure 6c), only time spent in the corridor was analyzed, and no 100 s criterion was imposed.

For measures of locomotor stability, we analyzed the portion of videos beginning with each animal's initial step until the completion of at least the fourth continuous step. Measures were calculated as follows: deviance from the center index (Figure 4b)—the sum of all paw distances (pixels) from the center of the corridor (Δ*Y*‐axis) after normalizing first to the number of steps, then to corridor width; coordination index—the average distance between where the forepaw lands in relation to the hindpaw's previous position (Δ*X*‐axis) for each side; paw precision index—average *r* ^2^ values derived from each paw's linear regression from start to finish of the task (Δ*X*‐ and Δ*Y*‐axes); total stride deviance index—standard deviation of absolute distances between step‐to‐step paw placements for each paw (Δ*X*‐axis); paw crossover rate—number of events when a paw crosses the midline to the opposite side divided by the number of steps.

### 4.8 Experimental design and statistical analysis

For all Ca^2+^ imaging experiments, the sample size needed to detect significance based on FOVs (power = 0.8; alpha = 0.05) was determined using SAS software (available online). Depending on the variable characterized, the minimum sample size given by the SAS power analysis ranged from 3 to 8 FOVs per group. As we typically obtain at least 2 FOVs per animal, we calculated that an *n* of 2 animals should be sufficient to yield power at or >80%. For most experiments, we typically imaged more than 25 FOVs per animal. However, in the Ca^2+^ imaging experiment using INS or INI delivery (Figure 2), fewer FOVs were imaged due to the repeated nature and time constraints associated with this experiment. For all gait experiments, sample sizes (*n* = 5–10 animals) were determined based on our prior experience (>30 years) performing behavioral characterizations in this animal model. To ensure a non‐biased investigation, we used the same rigorous statistical approach and filtering across all data. To address future reproducibility, we describe our methods in the greatest detail possible (e.g., reagent specifications, gait testing parameters, and imaging protocols used). Additionally, we find that our data aligns well with prior work in the literature showing increases in excitability with aging, providing here an independent form of replication. We report results from a total of 68 F344 animals. Of these, 24 were used for measures of the S1 network (young *n* = 10 animals [10,685 neurons]; aged *n* = 14 animals [11,395 neurons]) and 44 were used for measures of ambulatory performance (young *n* = 8; aged *n* = 36). For measures of Ca^2+^ network activity, a total of 7 FOVs (young *n* = 5; aged *n* = 2) were identified as outliers (Grubb's tests) based on baseline activity at rest and were removed from all subsequent analyses. No outliers were identified for measures of gait.

All statistical analyses were performed using SigmaPlot 14.0 (Systat Software Inc., San Jose, CA) and GraphPad Prism 9 (GraphPad Software Inc., San Diego, CA). Prior to testing for significance, datasets were first assessed for normality (Shapiro–Wilk tests) and equal variance (Brown–Forsythe tests). When normality was not satisfied using Student's *t* tests, a Mann–Whitney Rank Sum Test was performed instead (Figure 1e Overall Activity, Figure 3a Overall Activity and Synchronicity). Datasets undergoing ANOVA analysis that did not pass normality or variance assessments were either tested using a Welch's ANOVA (Figure 6a) or Log10 transformed prior to analysis (Figures 2a,c and 3a Connectivity and Connection Length). All other datasets were tested for significance using either Student's *t* tests (2‐sided) or ANOVAs (1‐way, 2‐way, 2‐way RM, or 3‐way RM) where appropriate. Overall, the data presented here are statistically robust and the main effects were unchanged irrespective of any corrections or data transformations performed. Significance for all measures was defined as *p* < 0.05. All data are presented as means ± standard error of the mean.

## AUTHOR CONTRIBUTIONS

Authors R‐L.L., H.N.F, K.L.A., S.L.C., A.O.G., and O.T. performed ambulatory behavior experiments. Authors K.L.A. and R‐L.L. performed data extraction and statistical analyses of ambulatory behavior. Author R‐L.L. performed 2P imaging experiments and statistical analyses. H.N.F. created and compiled the manuscript figures. Authors H.N.F., S.L.C., R‐L.L., and O.T. wrote and edited the manuscript text.

## CONFLICT OF INTEREST

The authors declare no competing financial interests.

## Data Availability

The datasets generated during and/or analyzed during the current study are available from the corresponding author on reasonable request. The authors will make all custom codes and algorithms used to extract data accessible upon request.
